# Yield, Fruit Quality, and Storability of ‘Canino’ Apricot in Response to Aminoethoxyvinylglycine, Salicylic Acid, and Chitosan

**DOI:** 10.3390/plants10091838

**Published:** 2021-09-04

**Authors:** Hayam M. Elmenofy, Sameh K. Okba, Abdel-Moety Salama, Shamel M. Alam-Eldein

**Affiliations:** 1 Fruit Handling Department, Horticulture Research Institute, Agricultural Research Center, Giza 12619, Egypt; dr.hmoustafa2015@gmail.com; 2 Deciduous Fruit Department, Horticulture Research Institute, Agricultural Research Center, Giza 12619, Egypt; bahshort@gmail.com; 3 Department of Horticulture, Faculty of Agriculture, Kafrelsheikh University, Kafr El-Sheikh 33516, Egypt; abdelmoaty.mohamed@agr.kfs.edu.eg; 4 Physiology and Breeding of Horticultural Crops Lab (PBHCL), Faculty of Agriculture, Kafrelsheikh University, Kafr El-Sheikh 33516, Egypt; 5 Department of Horticulture, Faculty of Agriculture, Tanta University, Tanta 31527, Egypt

**Keywords:** ethylene inhibitors, aminoethoxyvinylglycine, salicylic acid, chitosan, malondialdehyde, *PaACS1*, quality

## Abstract

Ethylene plays a pivotal role in the climacteric fruit ripening and senescence process. The effect of three ethylene inhibitors on the yield, quality, and storability of ‘Canino’ apricot fruit was studied. Foliar sprays of distilled water (control), aminoethoxyvinylglycine (AVG) (150 and 100 mg·L^−1^), salicylic acid (SA) (4 and 2 mM), and chitosan (2.5% and 1.5%) were applied 30 and 15 days before harvest. Results indicated that the high concentrations of AVG and SA recorded the lowest percentage of preharvest fruit drop and, hence, the highest yield. Trees receiving either concentration of AVG showed the highest fruit firmness. High concentrations of all three ethylene inhibitors reduced fruit weight loss, total carotenoids, and soluble solid content (SSC), but increased total acidity (TA) during cold storage (2 °C). A high score of overall taste acceptability was observed with a higher concentration of SA, which was also recorded the lowest fruit malondialdehyde content (MDA) at harvest and during storage. The highest concentrations of SA and chitosan recorded no decay for 28 days of storage. Gene expression analysis reflected higher expression of *PaACS1* gene with the highest concentrations of ethylene inhibitors, suggesting that SA (4 mM) is recommended for optimal yield, quality, and storability of ‘Canino’ apricot fruit grown under Egyptian conditions.

## 1. Introduction

Domesticated in China, apricot, *Prunus armeniaca* L., a member of the Rosaceae family, is one of the most widely distributed deciduous fruit trees in the world that produce highly nutritional fruit [1,2]. About 60% of the global production occurs in countries of the Mediterranean basin, with Egypt ranked first in productivity, but 11th in terms of the total cultivated area, which is about 6018 ha with a total annual production of 98,295,003 t (average of 16,333.5 t/ha) [3]. The climacteric nature of the fruit limits its shelf life to 3–5 days at ambient room temperature [4,5] and 4 weeks in cold storage (2 °C) [6]. The fruit marketability period is short due to high perishability; therefore, there is an exigency to develop methods that improve fruit quality and extend shelf life, along with improving overall orchard productivity through reduced preharvest fruit drop to get the utmost benefits of the cultivated area [7]. Preharvest applications have been used to control the fruit ripening and softening process due to ethylene synthesis [8]. Ethylene plays an important role in climacteric fruit ripening through ethylene signaling pathways [9], which are controlled by multigene-family-encoded enzymes 1-amino-cyclopropane-1-carboxylic acid synthase (ACS) (EC 4.4.1.14) and 1-amino-cyclopropane-1-carboxylic acid oxidase (ACO) (EC 1.14.17.4) [10]. Both enzymes increase ethylene biosynthesis, resulting in higher rates of respiration [11]. The higher rate of ethylene production improved the fruit ripening and senescence process and led to the formation of the abscission zone and, hence, fruit drop [12,13]. The ACS is a pyridoxal phosphate (PLP)-dependent enzyme that catalyzes the synthesis of 1-aminocyclopropane-1-carboxylic acid (ACC), a precursor for ethylene, from *S*-adenosyl methionine [14]. At the amino-acid level, the isoforms of ACS are biochemically evolving and acting in some specific cellular environments for ethylene biosynthesis. A phylogenetic tree was elaborated by comparing the C-terminal amino-acid sequences of three apricot ACS proteins with other ACS proteins from 10 different plant species [15]. Compared to plums, this analysis indicated that apricot ACS proteins could be divided into three main subfamilies: *P. armeniaca ACS1*, type 1, *P. armeniaca ACS2*, type 2, and *P. armeniaca ACS3*, type 3 [16]. Previous findings on ‘Patterson’ apricot showed that *ACS2* expression was significantly reduced with ethylene inhibition, suggesting its key role in ethylene biosynthesis during ripening. On the other hand, the expression of both *ACS1* and *ACS3* was higher with ethylene inhibition, which indicated that they were individually regulated in a specific way, as in other climacteric fruit [17].

Foliar application of plant biostimulants [18], potassium [5], abscisic acid (ABA) [19], chitosan, oligochitosan, and salicylic acid (SA) [20] effectively reduces the deterioration rate and improves apricot fruit quality and storability. Preharvest foliar applications to control fruit diseases using aminoethoxyvinylglycine (AVG) [21,22], SA [23,24], and chitosan [25,26] have also been claimed to diminish ethylene production. In this aspect, transcriptome testing (RNA sequencing) has been used as a valuable tool to study the molecular mechanism of fruit quality changes in apricot [2,27,28], persimmon [9], kiwifruit [29,30], pummelo [31], and banana fruit [32].

It has been reported that *ACS1* expression increased when ethylene was inhibited by AVG [33], indicating that the corresponding genes were individually regulated in a particular manner, as shown in pear [34]. Furthermore, it was noted that the effect of AVG on ethylene production rates is dose-dependent. For instance, application of AVG at 125 mg·L^−1^ decreased ethylene production rates and postponed the climacteric peak, while application at 250 mg·L^−1^ severely reduced ethylene production by 2–6-fold at the climacteric peak [34]. Previous reports have shown the role of AVG in reducing fruit drop, improving total yield [22], and maintaining the fruit quality of apricot [35], apple [22,36], sweet cherry [13], and pear [21]. 

The chorismate-derivative phytohormone, SA, is considered a natural phenolic acid [37] involved in plant growth and development [38]. It stimulates the defense mechanisms against various abiotic [24,39,40] and biotic stresses by inducing the accumulation of pathogenesis-related proteins [41]. Under stress conditions, SA has been shown to inhibit ethylene biosynthesis and its mode of action in plant [42] via the inhibition of the mitochondrial electron transport process [43], resulting in reduced respiration rate [11,44]. Therefore, SA has been proven to improve plant productivity, as well as maintain fruit quality and storability, when applied preharvest on plum [45,46], sweet cherry [47], and pomegranate [48]. It was also effective when applied postharvest, as reported on apricot [49,50,51].

Chitosan, a natural polysaccharide substance derived from the chitin of sea creatures, has been used to enhance the defense mechanism of plant and fruit against various types of biotic and abiotic stresses by altering stress-associated proteins such as heat-shock protein, disease resistance protein, and polyphenol oxidase, which are involved in cell-wall metabolism [52]. Previous findings revealed the effect of chitosan on fruit quality via reduced respiration and ethylene biosynthesis rates [26]. This effect was more pronounced with postharvest application of chitosan on apricot, mandarin, peach, apple, pomegranate, and guava [53,54,55,56,57,58]; however, very limited findings have been reported on its role in preharvest application [20,25,56]. 

In the Egyptian market, ‘Canino’ apricot is considered the latest-maturing apricot fruit with high remunerative value [5]. It also produces larger fruit than other cultivars; however, the fruit are very susceptible to chilling injuries and have limited shelf life [5]. The aim of this research was to improve fruit yield, quality, shelf life and marketability through an evaluation of the effect of preharvest foliar application of some ethylene inhibitors (e.g., AVG, SA, and chitosan) on fruit characteristics at harvest and during cold storage.

## 2. Results and Discussion

### 2.1. Preharvest Fruit Drop and Total Yield 

**Figure 1:**
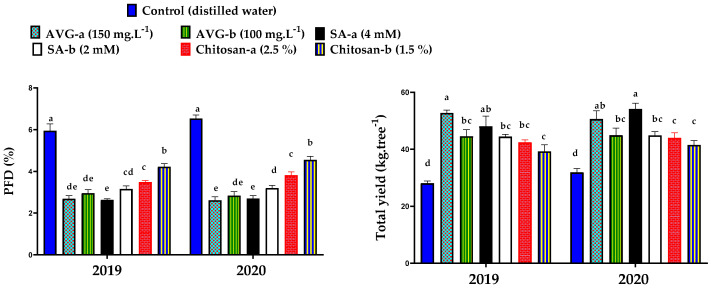
Effect of preharvest foliar application of aminoethoxyvinylglycine (AVG), salicylic acid (SA), and chitosan on preharvest fruit drop (PFD) and total yield of ‘Canino’ apricot trees during the 2019 and 2020 seasons. Values are the mean ± the standard deviation (SD). Means followed by the same letters are not significantly different using DMRT at *p* ≤ 0.05.

Foliar application of AVG, SA, and chitosan significantly reduced the percentage of preharvest fruit drop (PFD) and, consequently, increased the yield of ‘Canino’ apricot trees in comparison to the untreated trees (control), as shown in Figure 1. The high concentration of AVG (150 mg·L^−1^), followed by SA (4 mM), recorded the lowest percentage of PFD, along with the highest yield, compared to the remaining treatments and the control in the 2019 and 2020 seasons. The control recorded the highest PFD and the lowest yield. Preharvest fruit drop could be minimized with a delayed fruit maturity rate using plant bioregulators that inhibit ethylene biosynthesis [22]. Ethylene has an effect on indole acetic acid (IAA) depletion that is thought to increase the response of the abscission zone cells to enzymatic signals that stimulate cellular breakdown. These enzymes are like cellulase and polygalacturonase, which dissolve the cell wall, accelerate fruit maturity, and eventually induce fruit abscission [59]. Previous findings revealed that PFD is diminished with delayed fruit ripening using AVG that reduced ethylene biosynthesis via the inhibition of ACC synthase activity [22,60,61]. It was reported that spraying apple trees with AVG four weeks before harvest delayed the fruit ripening rate and decreased PFD by 50% [12]. Preharvest application of SA also decreased PFD [62]. Chitosan slowed the cell structural degradation in the abscission zone by diminishing the function of cell-wall-degrading enzymes [63]. 

### 2.2. Weight Loss and Fruit Firmness

**Figure 2:**
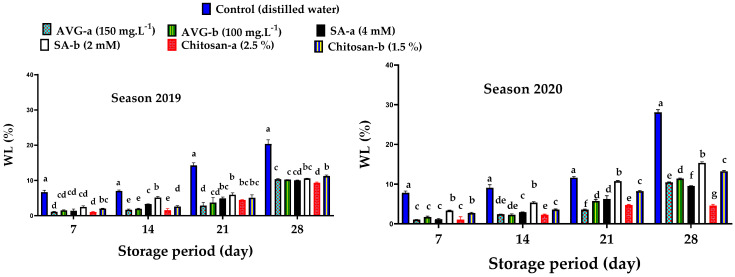
Effect of preharvest foliar application of AVG, SA, and chitosan on fruit weight loss (WL) of ‘Canino’ apricot after 7, 14, 21, and 28 days of storage at 0 °C and 90% RH during the 2019 and 2020 seasons. Values are the mean ± SD. Means followed by the same letters are not significantly different using DMRT at *p* ≤ 0.05.

At harvest, fruit weight loss (WL) was the highest in control fruit, compared to other treatments. In general, WL percentage significantly increased with the prolonged storage, regardless of the treatment (Figure 2). The control recorded the highest WL percentage, but the lowest percentages were recorded for chitosan (2.5%), followed by SA (4 mM)), after 28 days of storage in both seasons. 

**Figure 3:**
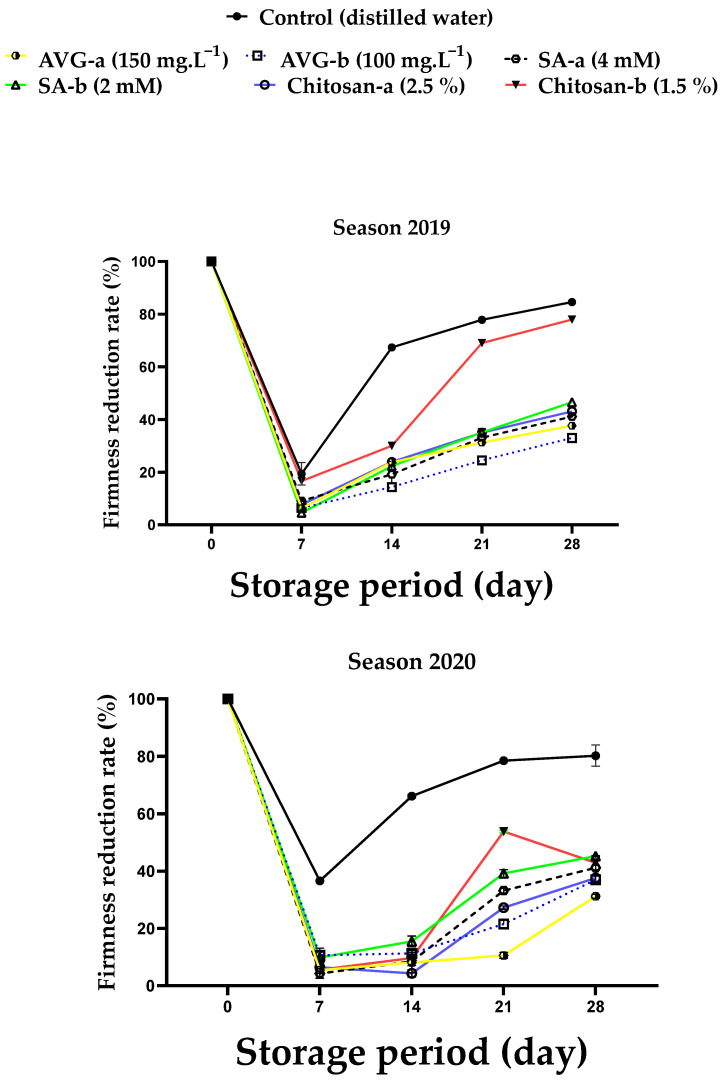
Effect of preharvest foliar application of AVG, SA, and chitosan on fruit firmness reduction rate of ‘Canino’ apricot after 7, 14, 21, and 28 days of storage at 0 °C and 90% RH, followed by 2 days of shelf life at 20 ± 2 °C and 80–85% RH during the 2019 and 2020 seasons. Values are the mean ± SD.

The firmness reduction rate (fruit softening rate) increased with the prolonged storage in comparison to harvest date, with the highest reduction in firmness recorded for the control fruit during both seasons (Figure 3). The application of AVG, SA, and chitosan significantly delayed fruit softening, particularly at the higher concentrations, with the most pronounced effect recorded for AVG, followed by SA, and then chitosan. The positive effect of these compounds on fruit firmness could be due to the reduction in ethylene biosynthesis and the reduced activity of polygalacturonase and pectin methylesterase responsible for the degradation of polysaccharides in fruit cell walls leading to fruit softening [64].

Increased levels of WL during storage could also be a result of increased rates of respiration [65]. Previous reports have also indicated that AVG improved fruit firmness in sweet cherry [13], pear [21], and apple [12,22]. In addition, SA-treated fruit recorded a small reduction in WL (Figure 2), which was associated with improved fruit firmness (Figure 3). These results are supported by the previous reports on sweet cherry [45] and plum [46]. Chitosan-treated grapes showed a reduction in fruit water loss due to increased cell-wall stabilization through the accumulation of lignin and/or the formation of cross-linked hydrogen bonds between chitosan and lignin, which resulted in the creation of a firm network structure on fruit surface, leading to a preserved cell-wall structure and stability that was reflected on improved berry firmness [26,63]. Likewise, chitosan-coated mango fruit have shown a reduction in WL due to the covered stomata on fruit peel that led to reduced rates of transpiration and respiration [66].

### 2.3. Decay Incidence

**Figure 4:**
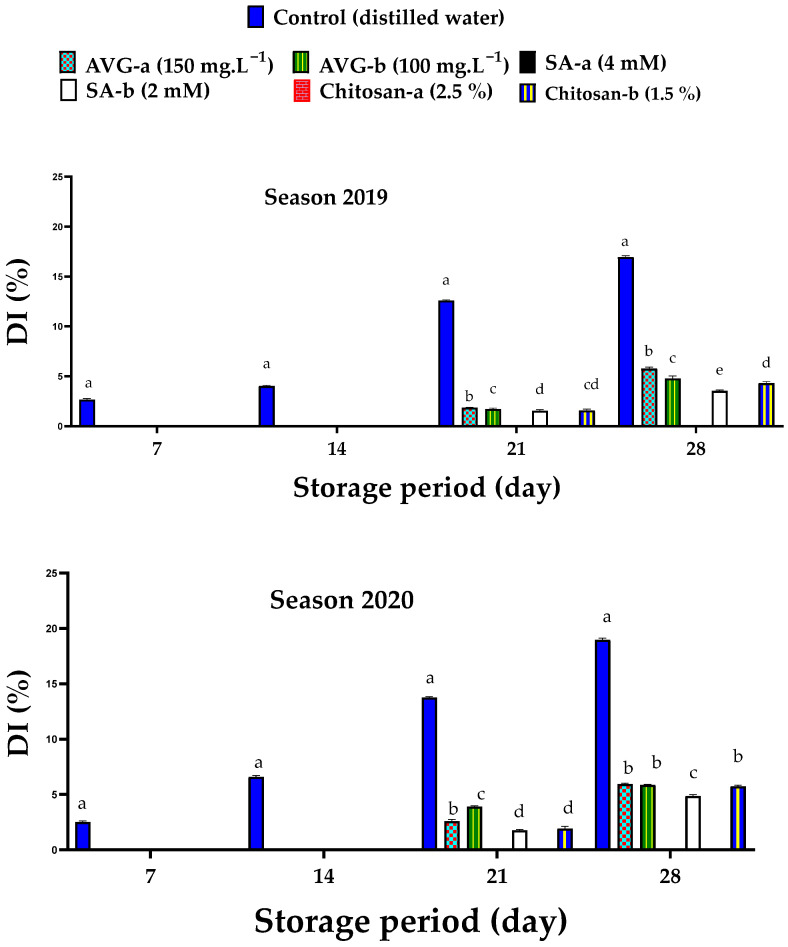
Effect of preharvest foliar application of AVG, SA, and chitosan on fruit decay incidence (DI) of ‘Canino’ apricot after 7, 14, 21, and 28 days of storage at 0 °C and 90% RH, followed by 2 days of shelf life at 20 ± 2 °C and 80–85% RH during the 2019 and 2020 seasons. Values are the mean ± SD. Means followed by the same letters are not significantly different using DMRT at *p* ≤ 0.05.

Control fruit showed an increased percentage of decay incidence (DI) with prolonged storage period (Figure 4); however, ethylene inhibitors substantially diminished fruit DI, which started by the 21st day of storage in AVG-treated fruit (both concentrations), followed by lower values for chitosan- and then SA-treated fruit (lower concentrations only), while fruit treated with the higher concentrations of SA and chitosan showed no DI by the end of storage. Interestingly, there was a positive correlation between DI and WL (*r* = 0.939 *** and 0.944 ***) by the end of storage in both 2019 and 2020 seasons, respectively. Fruit susceptibility to postharvest pathogens generally increases as the peel softens with maturation and senescence; therefore, less force is required to invade the peel [67]. Mechanical injuries during harvest and handling are the main sites of peel invasion by pathogens [68]. High humidity during storage is important to maintain peel resistance against pathogens [69]. Low temperature noticeably retards the growth of pathogens on the fruit surface; however, disease symptoms appear when infected fruit are transferred to warm temperature [70]. 

The high concentrations of SA and chitosan have substantially preserved fruit quality (Figure 4). The role of SA could involve triggering the fruit’s local and systemic resistance to pathogens and their related proteins or polyphenols [62]. In addition, SA has a positive impact on the plant antioxidant system, as well as the phenylpropanoid metabolism cycle and its related genes, resulting in fruit rot inhibition [24]. It was also reported that SA affected DELLA proteins (class of nuclear growth-repressing proteins) that protect cells from a wide range of pathogens [71]. Preharvest application of SA on sweet cherry induced plant antioxidant capacity and improved the biosynthesis of phenols and anthocyanins, as well as the activity of catalase (CAT), peroxidase (POD), ascorbate peroxidase (APX), and superoxide dismutase (SOD), which protect cells against the generated free radicals [47]. The anti-senescent effect of SA was reported to maintain apricot fruit firmness and eventually reduce DI [20,72,73]. A negative correlation between fruit antioxidant activity and fruit decay was noticed [50]. Previous findings indicated that SA or chitosan could delay the rate of apricot fruit deterioration during storage by inducing the activity of defense-related enzymes (e.g., chitinase and β-1,3-glucanase) and preserving the bioactivity and antioxidant ability of phenol compounds. The charged groups of the chitosan polymer and their ionic interactions with the components of the bacterial cell wall can cause bacterial death and protect fruit from the infectious agents [74,75,76,77]. Chitosan also formed a thick film on the fruit surface, preventing the penetration of pathogen hyphae, and extended the storage period of tangerine [78]. Similar results were reported on apricot [20], mango [79], and guava [80].

### 2.4. Fruit Color and Total Carotenoids 

The loss in green color is mainly related to ethylene generation during ripening, which activates chlorophyll oxidase that breaks chlorophyll pigments [80]. Preharvest application of ethylene inhibitors effectively delayed fruit color development at harvest compared to the control fruit during the 2019 and 2020 seasons (Table 1). The best result was recorded for AVG at high concentration. Consequently, fruit color development during storage was slower than that of the control fruit. The most remarkable effect was seen for SA (both concentrations) and chitosan (high concentration), as indicated by the changes in color parameters (L*—lightness, a*—red/green, and b*—yellow/blue) after 28 days of storage in comparison to the control. The role of SA and chitosan in retarding color development has previously been confirmed on cherry and apple fruit, respectively [47,56]. It could be suggested that the higher total color difference (ΔE) of the SA and chitosan treatments, compared to the control, might has been due to the higher L* values at 28 days, because of the less developed color (i.e., greater luminosity), while L* values were lower for the well-developed and dense color of the control fruit (i.e., the least luminosity). In addition, the AVG-treated fruit were the most green ones, based on the values of a* and b*; however, the lower L* values in the control fruit after 28 days of storage were due to the darker green color (compared to SA- and chitosan-treated fruit) that reduced overall fruit luminosity, but the lower ΔE in this case (compared to the control) was due to the negative a* values at harvest. These results are supported by previously reported findings on mango [81].

**Figure 5:**
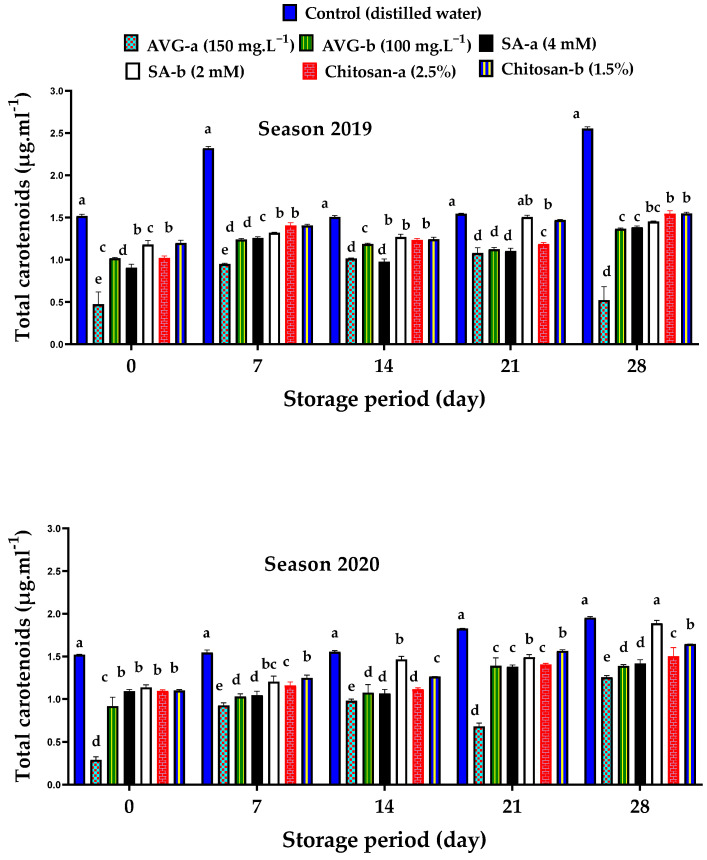
Effect of preharvest foliar application of AVG, SA, and chitosan on total carotenoids of ‘Canino’ apricot fruit at harvest (0 day), and after 7, 14, 21, and 28 days of storage at 0 °C and 90% RH, followed by 2 days of shelf life at 20 ± 2 °C and 80–85% RH during the 2019 and 2020 seasons. Values are the mean ± SD. Means followed by the same letters are not significantly different using DMRT at *p* ≤ 0.05.

The distinct color of apricot fruit is mainly related to the carotenoid pigments [82]. At harvest, the total carotenoid contents of the ethylene-inhibitor-treated fruit were substantially lower than that of the control fruit (Figure 5). Overall, carotenoid contents steadily increased throughout the storage in both treated and control fruit; however, the control showed the highest content by the end of the storage period. All ethylene inhibitors effectively retarded the deterioration in chlorophyll pigments that emasculate the carotenoid pigments, thereby delaying color development by the end of the storage. The most pronounced effect in this regard was recorded for the higher concentration of AVG during both seasons. During ripening, climacteric fruit showed an increase in fruit color development with increased ethylene levels that mainly activate chlorophyll oxidase, responsible for chlorophyll degradation [80]. Previous findings showed that the application of AVG, SA, or chitosan effectively delayed the ethylene biosynthesis and, hence, fruit ripening and color development [6,20,22].

**Figure 6:**
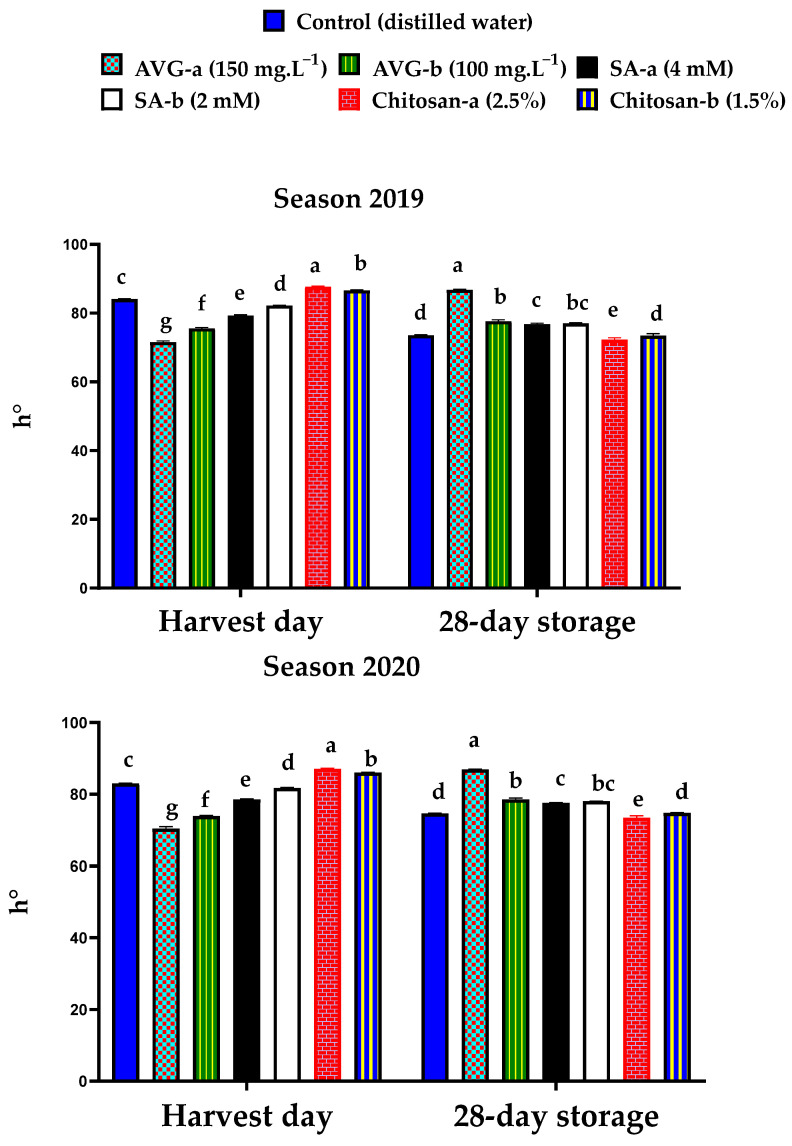
Effect of preharvest foliar application of AVG, SA, and chitosan on hue angle (h^0^) of ‘Canino’ apricot fruit at harvest and after 28 days of storage at 0 °C and 90% RH, followed by 2 days of shelf life at 20 ± 2 °C and 80–85% RH during the 2019 and 2020 seasons. Values are the mean ± SD. Means followed by the same letters are not significantly different using DMRT at *p* ≤ 0.05.

The hue angle (h^0^) presented in Figure 6 is another parameter for color index. ‘Canino’ apricot fruit were harvested at a yellowish-green stage with higher degrees of h^0^ (>80) for the chitosan-a, chitosan-b, and control treatments, compared to the other treatments. Previous findings recorded h^0^ values over 75 at harvest for ethylene-inhibitor-treated ‘Modesto’ and ‘Patterson’ apricot fruit [83]. Higher h^0^ values were recorded by the end of the storage for the AVG-a-treated fruit, followed by the AVG-b and SA-b, and then SA-a, in comparison to the chitosan-a, chitosan-b, and control treatments. The higher h^0^ values by the end of the storage (Figure 6) reflected the reduction in fruit ripening, associated with lower carotenoid contents (Figure 5). There was a negative correlation between h^0^ and total carotenoids by the end of the storage (*r* = −0.785 *** and −0.567**) in the 2019 and 2020 seasons, respectively. This negative correlation was previously confirmed in 37 apricot cultivars [84].

### 2.5. Soluble Solid Content, Total Acidity, and Ripening Index 

Data presented in Table 2, Table 3 and Table 4 respectively revealed that control fruit recorded the highest soluble solid content (SSC), but the lowest total acidity (TA) and, therefore, the highest ripening index (RI), in comparison to all other treatments during both seasons. As a climacteric fruit, apricot generally showed an increase in SSC and RI, associated with a reduction in TA during storage. Increased ethylene levels with the ripening process resulted in the accumulation of glucose, fructose, and sucrose [85], associated with reduced TA due to the depletion of organic acids in cell respiration (tricarboxylic acids cycle) [86]. The reduction in fruit water content with prolonged storage, along with the reduction in TA, could also have been a possible reason for the gradual increase in free sugars during storage [65,87]. Increased fruit sugar contents are usually associated with improved SSC and, consequently, improved fruit RI. As the fruit ripens, ethylene induces the biosynthesis of phenolic compounds, which have been shown to be the major influence on the sensory quality of the fruit (e.g., color, flavor, and taste) [88,89]. 

All ethylene-inhibitor-treated fruit recorded lower values of SSC and RI, associated with higher values of TA, compared to the control at harvest date, as well as by the end of the storage period during both seasons. The most remarkable effect was recorded with the high concentration of AVG (Table 2, Table 3 and Table 4). In this regard, the applications of SA and chitosan were generally the second and third most effective treatments, compared to the control. It has been reported that the application of AVG significantly delayed starch degradation and reduced SSC, but enhanced acidity in sweet cherry [13], pear [21,34], and apple [12,22]. Similarly, SA was proven to delay the ripening and senescence process of plum [46], apricot [20,49], and mango fruit [40], represented by low SSC and high organic acid contents. Chitosan was also effective in improving the postharvest fruit quality of apricot [20], raspberry [25], and pomegranate [57]. It reduced the conversion of protopectins into water-soluble pectins and, therefore, slightly improved SSC in stored ‘Kinnow’ mandarin fruit, as a result of a slowed rate of senescence with prolonged storage [54]. 

### 2.6. Sensory Analysis

**Figure 7:**
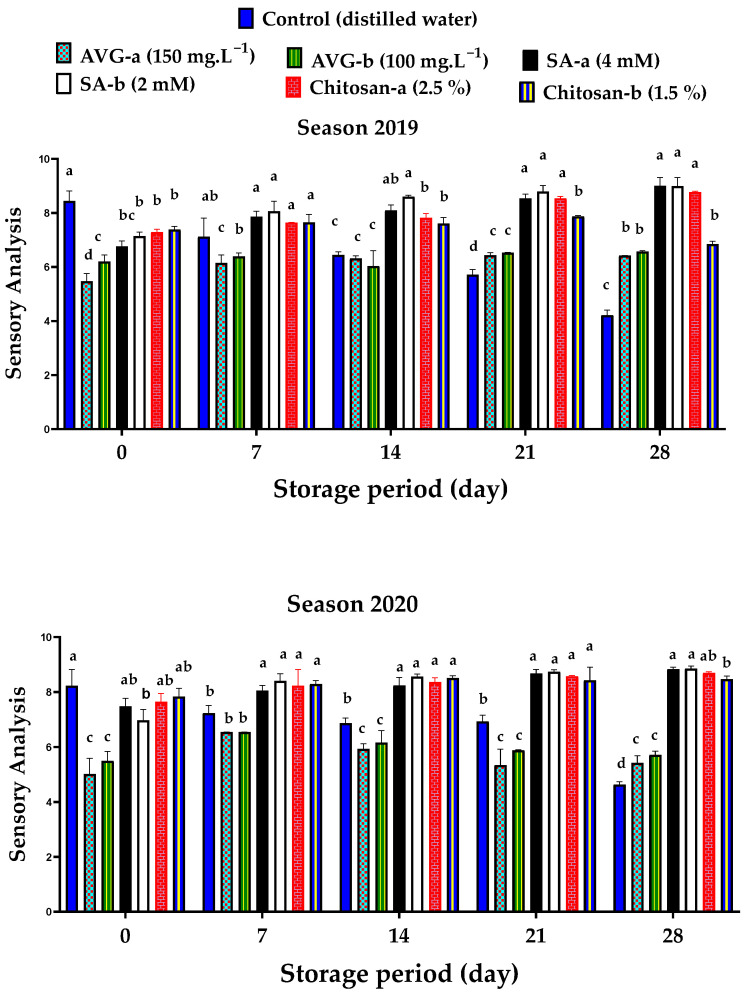
Effect of preharvest foliar application of AVG, SA, and chitosan on the sensory analysis of ‘Canino’ apricot fruit at harvest (0 day) and after 7, 14, 21, and 28 days of storage at 0 °C and 90% RH, followed by 2 days of shelf life at 20 ± 2 °C and 80–85% RH during the 2019 and 2020 seasons. Values are the mean ± SD. Means followed by the same letters are not significantly different using DMRT at *p* ≤ 0.05.

The sensory analysis of ‘Canino’ apricot fruit is an overall assessment of fruit firmness (Figure 3), color (Table 1), and taste (Table 4). Sensory analysis indicated that the control fruit was the best at harvest, but lost its overall customer acceptance with the prolonged storage during both seasons. On the other hand, the ethylene-inhibitor-treated fruit showed lower acceptability at harvest, but overall acceptability improved with prolonged storage, except for AVG-treated fruit, which remained less acceptable by the end of the storage period (28 days) (Figure 7). At harvest, the control fruit received the best scores (8.44 ± 0.38 and 8.23 ± 0.6) in both 2019 and 2020 seasons, respectively. However, by the end of the storage period, almost similar values were reported for the SA-a- (9.00 ± 0.31 and 8.83 ± 0.08), SA-b- (8.99 ± 0.33 and 8.86 ± 0.09), and chitosan-a-treated fruit (8.77 ± 0.03 and 8.71 ± 0.03) in both 2019 and 2020 seasons, respectively. This could be related to the enhanced, but delayed ripening process of the control and ethylene-inhibitor-treated fruit, respectively [6]. Both SA and chitosan could effectively delay ripening and maintain overall fruit appearance during storage, thus extending fruit marketability with overall good quality [20]. The application of AVG was the most effective in delaying fruit ripening throughout the storage period (Figure 7), thus suggesting the potential for a longer storage period and, hence, a longer marketability period, compared to the SA and chitosan treatments. However, it all depends on the trend of overall fruit WL percentage and susceptibility to DI after 28 days (which can be considered in future research). 

### 2.7. Lipid Peroxidation 

**Figure 8:**
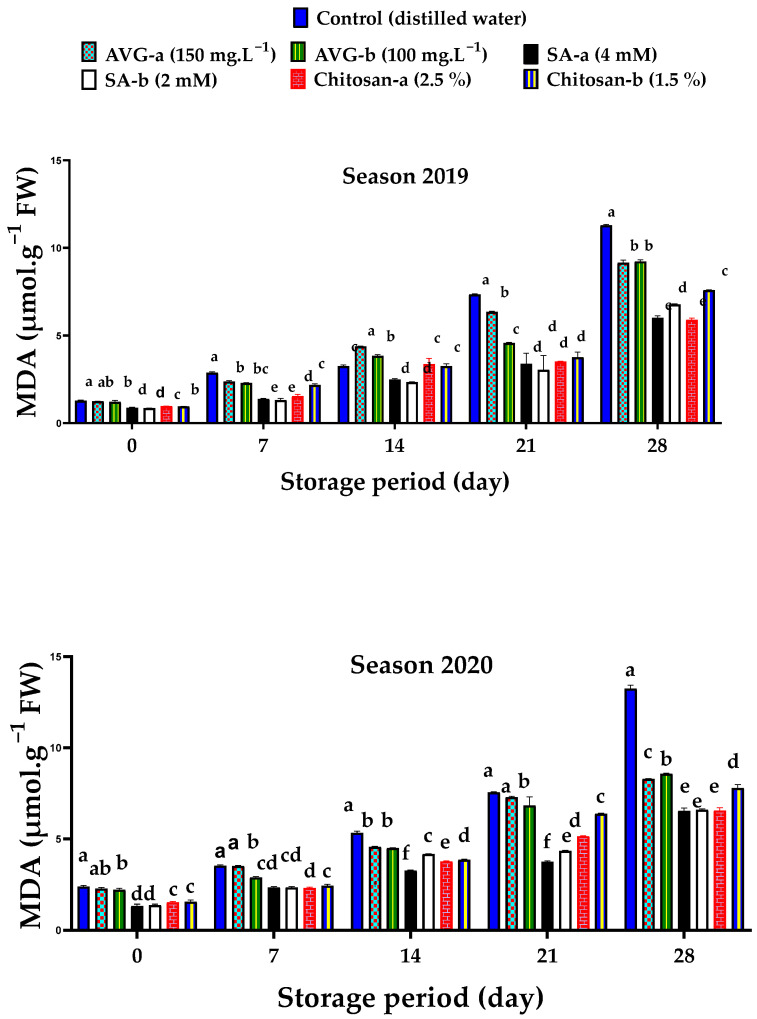
Effect of preharvest foliar application of AVG, SA, and chitosan on the malondialdehyde (MDA) content of ‘Canino’ apricot fruit at harvest (0 day) and after 7, 14, 21, and 28 days of storage at 0 °C and 90% RH, followed by 2 days of shelf life at 20 ± 2 °C and 80–85% RH during the 2019 and 2020 seasons. Values are the mean ± SD. Means followed by the same letters are not significantly different using DMRT at *p* ≤ 0.05.

Malondialdehyde (MDA) is the final product of lipid oxidation; thus, it can be used as indicator of lipid peroxidation of the cellular membrane [90]. Results indicated that both the control and AVG-a-treated fruit showed the highest concentration of MDA, compared to the other treatments at harvest date of both seasons. Both concentrations of SA recorded the lowest MDA contents (Figure 8). In general, MDA content increased with prolonged storage. Both SA and chitosan treatments successfully reduced MDA levels in stored fruit, whereas AVG and the control increased its levels. The highest and the lowest MDA contents were observed for the control and the highest concentration of SA, respectively. The effect of SA and chitosan on MDA content has previously been reported on apricot. Both components improve plant antioxidant capacity and prevent cell-wall degradation through their positive effect on phenols and antioxidant enzymes [20]. It has been reported that SA effectively increases melatonin content, which has an antioxidant function, as a scavenging ROS, reducing membrane lipid peroxidation [62]. In addition, SA effectively improved the expression of cytosolic malate dehydrogenase and strengthened the plant cell’s redox state in apple [91] Chitosan protected membrane integrity by limiting the lipoxygenase activity and MDA accumulation [92].

### 2.8. PaACS1 Gene Expression

**Figure 9:**
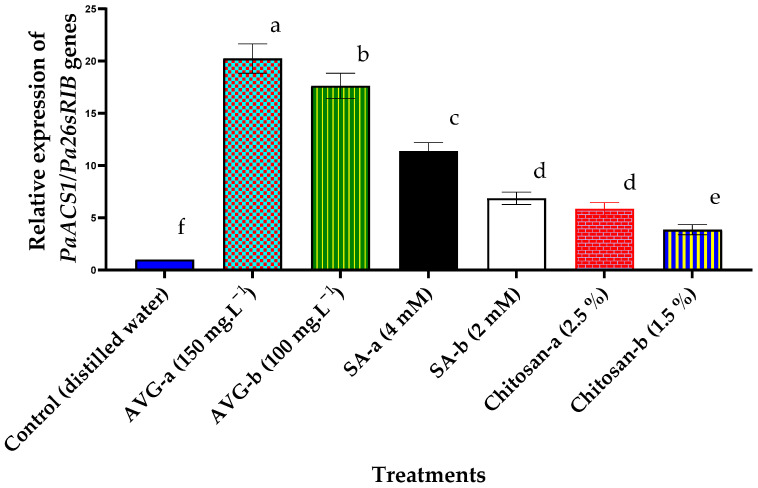
Effect of preharvest foliar application of AVG, SA, and chitosan on the ripening-related *PaACS1* gene expression in ‘Canino’ apricot fruit at harvest during the 2019 and 2020 seasons. Gene expression was normalized against the housekeeping gene *Pa26sRIB*. Values are the mean of both seasons ± SD (*n* = 10). Means followed by the same letters are not significantly different using DMRT at *p* ≤ 0.05.

With the exception of housekeeping genes required for basic cellular functions, gene expression could be defined as a biological process that varies in response to environmental stimuli, eventually affecting the plant’s response to its surroundings [52]. Results of the quantitative real-time polymerase chain reaction (qRT-PCR) revealed a significant increase in the transcription levels of *PaACS1* gene in fruit treated with AVG, SA, or chitosan, in comparison to the control. The most conspicuous effect was noted with AVG, followed by SA, and then chitosan, with a remarkable effect seen for the high concentration of each compound (Figure 9). These findings are consistent with the previous reports on the role of ethylene inhibitors in ripening-related gene expression [17,27,28,33,64] with an emphasis on chitosan [25] and SA [91]. 

The present findings revealed that AVG, SA, and chitosan effectively retarded the preharvest maturation process and delayed ‘Canino’ apricot fruit deterioration during storage. In this regard, AVG and SA effectively reduced PFD and improved total yield (Figure 1), but AVG was more effective in reducing the fruit softening rate (Figure 3), color development (Table 1 and Figure 6), carotenoid contents (Figure 5), SSC (Table 2), and ripening index (Table 4), whereas SA followed by chitosan recorded the lowest DI (Figure 4), TA (Table 3), MDA (Figure 8) and *PaACS1* gene expression (Figure 9). The lowest values of WL (Figure 2) were recorded with chitosan treatments. In terms of the used concentration, some very few variations among these three compounds in some parameters including sensory analysis (Figure 7) were also noticed. Overall, the best results on fruit behavior during storage were related to the high concentrations of SA, followed by chitosan. Although AVG was the best inhibitor of ethylene, the delayed maturation at harvest was the reason for the low quality of fruit following the 28 days of cold storage, represented by the uncommon preserved green color and increased percentage of WL, compared to SA and chitosan treatments. These results are consistent with previous findings reported with peach [93]. It was also reported that cell division occurs very quickly in early harvested cultivars, which may be associated with uncompleted cell growth at harvest. Therefore, the respiration rate of the fruit is quite high with excessive water loss due to the less-formed peel structure, leading to reduced fruit weight [94]. In addition, AVG could affect fruit maturity at harvest, as well as modify the link between visual maturity factors such as fruit color and other maturity factors such as firmness and SSC [95]. This could explain the high values of fruit firmness along with the low values of fruit color and SSC with AVG treatments. Moreover, the uncompleted cell growth could also be another reason for the fruit’s susceptibility to chilling injury, which could be associated with the increased level of DI [96], as shown in the AVG-treated fruit, compared to SA and chitosan treatments, by the end of the storage period. Therefore, the AVG treatment eventually resulted in unmarketable fruit by the end of the cold storage period; hence, SA treatment at the higher concentration (4 mM) was found to be more effective in this regard.

## 3. Materials and Methods

### 3.1. Experiment

This experiment was carried out on 8-year-old ‘Canino’ apricot trees (*Prunus armeniaca* L.) grown in a private orchard located at Nubaria district, Beheira Governorate (30°69′91″ N, 30°66′86″ E), Egypt, during the 2019 and 2020 seasons. A total of 42 apricot trees grafted on seedling rootstocks of ‘Canino’ apricot, planted at 4 × 5 m spacing in sandy soil, similar in size and vigor with no symptoms of nutrient deficiency, were selected for this experiment. Trees were subjected to drip irrigation and the same agricultural practices as the entire orchard, and they were distributed in a randomized complete block design [97] of seven treatments with three replicates each. Two trees represented each replicate. Soil analysis is displayed in Table 5.

Seven foliar spraying treatments were applied twice at 30 and 15 days before harvest, as follows: distilled water (control), aminoethoxyvinylglycine (AVG) at 150 and 100 mg·L^−1^, salicylic acid (SA) at 4 and 2 mM, and chitosan at 2.5% and 1.5%. Aminoethoxyvinylglycine was prepared from ‘ReTain’ [15% active ingredient] (Valent BioScience Corporation, Libertyville, IL, USA). Chitosan (C_6_H_11_NO_4_; 100–300 MW) (Cornell Lab, Cairo, Egypt) was prepared according to Tezotto-Uliana et al. [25]. All solutions of AVG, SA (Oxford Laboratory Reagents, Mumbai, India), and chitosan were prepared using distilled water and mixed with Tween 20 (0.5% *v*/*v*) as a surfactant for a total volume of 4 L per tree.

### 3.2. Studied Parameters 

Preharvest fruit drop (PFD) was determined daily by counting the number of dropped fruit under each tree for six consecutive days before harvest, and then PFD was calculated as a percentage in relation to the total number of harvested fruit [60]. Fruit were harvested at the yellowish-green stage by the end of the first week of June during both seasons (~70–80 days from full bloom) [88]. Fruit were packaged in commercial plastic containers and promptly transported to the laboratory. Fruit were then washed with tap water mixed with chlorine (1 mg·L^−1^) with no pesticide or waxing treatments, and left for air-drying at room temperature (22–23 °C) for 30 min.

A sample of 10 uniform fruits per tree (20 fruits/replicate) was randomly collected and weighed using a bench-top digital scale Model PC-500 (Doran scales, Inc., Batavia, IL, USA) with an accuracy of 0.1%. Average fruit weight (g) was calculated, and then multiplied by the total number of fruit per tree to calculate total fruit yield (kg·tree^−1^). The same fruit samples were used for harvest date analyses. 

Another four samples, 20 fruits each per replicate (60 fruits/treatment), were collected free of mechanical injuries and decay. Each group of fruit was placed in a cardboard box and stored at 0 °C and 90% RH. At weekly intervals (i.e., 7, 14, 21, and 28 days), one box was used to evaluate fruit characteristics during storage. Fruit WL (%) was calculated according to the following equation:

Weight loss (WL) (%) = ((fruit weight at harvest − fruit weight after storage)/fruit weight at harvest) × 100.(1)

After storage, fruit were placed at room conditions (20 ± 2 °C and 80–85% RH) for 2 days, as a shelf-life period, before assessing other fruit parameters. Average fruit firmness (Newton/cm^2^) at harvest and at weekly intervals during storage was measured at the equatorial area on two sides of 10 fruits using a handheld Shimpo digital force gauge, Model FGV-50XY fitted with 8 mm diameter plunger tip (Shimpo company, Wilmington, NC, USA). The firmness reduction rate (fruit softening rate) during each storage period was calculated as a percentage of the original fruit firmness at harvest using the following equation:

Firmness reduction rate (%) = ((fruit firmness at harvest − fruit firmness after storage)/fruit firmness at harvest) × 100.(2)

The number of decayed fruit was also counted at weekly intervals, and decay incidence was calculated as a percentage of the original fruit number, as follows:

Decay Incidence (DI) (%) = (number of decayed fruit/total number of fruit) × 100.(3)

Fruit color was colorimetrically assessed on two opposite sides at the equatorial area of each fruit [98] using a Minolta colorimeter (Minolta Co. Ltd., Osaka, Japan), and the color was recorded according to the Commission Internationale d’Eclairage L*, a*, and b* (CIELAB color system) that represents a uniform three-dimensional color space coordinates, where L* is the lightness coordinate (dark-bright scale), a* is the red/green coordinate (with +a* for red color, and −a* for green color), and b* is the yellow/blue coordinate (with +b* for yellow color, and −b* for blue color) [98]. Hue angle (h^0^ = tan^−1^(b*/a*) was also calculated at harvest and by the end of the storage period. Color was assessed after a period of cold storage plus 2 days at room conditions, in comparison to fruit color at harvest date to calculate total color difference (ΔE), using the following formula [99]:

ΔE = (ΔL^2^ + Δa^2^ + Δb^2^)^1/2^, (4)

where ΔL, Δa, and Δb represent the differences in L, a, and b values, respectively.

Fruit chlorophyll and carotenoid contents (µg·mL^−1^) were measured at harvest and then at 7-day intervals during storage [100]. Five grams of fruit sample was dissolved in 30 mL of 80% acetone and measured using a spectrophotometer (UV/visible spectrophotometer Libra SS0PC, Thermo Fisher Scientific, Waltham, MA, USA). The absorbance was recorded at 663, 646, and 470 nm for chlorophyll a, chlorophyll b, and carotenoids, respectively, and total contents were calculated according to the following equations:

Chlorophyll (a) = 12.21 E663 − 2.81 E646,(5)

Chlorophyll (b) = 20.13 E646 − 5.03 E663,(6)

Total carotenoids = ((1000 E470) − (3.27 × chlorophyll a + 104 × chlorophyll b))/198,(7)

where E is the optical density at the specified wavelength. 

Extracted juice from about 200 g of fruit was used to determine SSC (%), using a digital refractometer (RFM 340-T, KEM Kyoto Electronics Manufacturing Co. Ltd., Tokyo, Japan). Total acidity (TA) was estimated as malic acid (%) [101] using an automated titration device (TitroLine, TL 5000, SI Analytics, Weiheim, Germany). Fruit RI was expressed as the SSC/TA ratio.

A group of eight trained panelists ran a test panel to evaluate fruit sensory attributes (e.g., general appearance, texture, color, and taste) using a hedonic scale of 1–9 for each sensory attribute (excellent (9), very good to excellent (8), very good (7), good to very good (6), good (5), average (4), acceptable (3), unsatisfactory to acceptable (2), and unsatisfactory (1)) [102]. Scores were then averaged, and a score ≥ 5 was considered acceptable for commercial purposes [49]. 

Lipid peroxidation of the cellular membrane was determined by estimating the MDA concentration using thiobarbituric acid-reactive substances (TBARS) [103,104] with some modifications, where a sample of fresh tissue (0.4 g) was homogenized in 20 mL of 10% trichloroacetic acid (TCA) and centrifuged at 10,000× *g* for 10 min. The supernatant (2 mL) was mixed with 2 mL of 2-thiobarbituric acid (TBA) (0.5%) in a test tube, and then heated at 95 °C for 15 min in a water bath. The tube was immediately cooled in ice bath, and then centrifuged at 1800× *g* for 10 min. The solution was then tested using a spectrophotometer (UV/visible spectrophotometer Libra SS0PC, Thermo Fisher Scientific, Waltham, MA, USA) at wavelengths of 450, 532, and 600 nm, and the amount of accumulated MDA was calculated as follows:

MDA (μmol·g^−1^ FW) = ((6.452 (OD532 − OD600) − 0.559 OD450) × 10 mL)/FW, (8)

where FW is the fresh weight of the fruit sample (g).

The ripening-related *PaACS1* gene expression analysis was performed by the qRT-PCR [16]. Total RNA was extracted using a Gene JET RNA Purification Kit # K0731 (Thermo Fisher Scientific, Waltham, MA, USA). The cDNA was synthesized by reverse transcription using the RevertAid H Minus Reverse Transcriptase kit # EP0451 (Thermo Fisher Scientific, Waltham, MA, USA). The qRT-PCR mixture included cDNA, Syber green master mix (2X Maxima kit # K0221, Thermo Fisher Scientific, Waltham, MA, USA), and primers, while the β-actin gene was used as a reference (internal control). The primers were designed using the Primer 3 web-based tool on the basis of the apricot sequence retrieved from the gene bank database. The thermal cycling and melting curve conditions were performed as previously described by El-Adawy et al. [105]. The relative gene expression was presented as an average of both seasons in terms of fold change using the 2^−∆∆Ct^ method [106], according to the following primers:

*PaACS1*(f) 5′–ATTCAACCAGGCAAAGAAACGC–3′,
*PaACS1*(r) 5′–GATGGAGTGGAAATGGACGAGA–3′,
*Pa26sRIB*(f) 5′–AACGCAGGTGTCCTAAGATGAG–3′,
*Pa26sRIB*(r) 5′–GCTGCCACAAGCCAGTTATCC–3′.

### 3.3. Statistical Analysis

Data were first analyzed for numerical normality and homogeneity of variance using Shapiro–Wilk’s and Levene’s tests, respectively. Data were then statistically analyzed, and one-way analysis of variance (ANOVA) was performed using CoStat software package (version 6.303, Monterey, CA, USA). Means were expressed as the value ± standard deviation (SD) and compared using Duncan’s multiple range test (DMRT) at *p* ≤ 0.05 [107]. 

## 4. Conclusions

Managing the appearance of ‘Canino’ apricot fruit in the market, as well as extending fruit shelf life with minimal negative effects on yield and fruit quality, is a crucial issue in the Egyptian apricot industry. To address this, we carried out an investigation that revealed that the foliar spray of ethylene inhibitors such as AVG, SA, and chitosan reduced the percentage of PFD compared to the control, with the highest yield recorded for both AVG and SA. The effect of AVG on fruit firmness was more pronounced in comparison to SA and chitosan. All three compounds were effective in reducing fruit WL, total carotenoids, and SSC, along with increased TA in comparison to the control during the 28 days of cold storage at 0 °C. Fruit sensory analysis reflected the highest quality with the application of SA. In addition, this treatment also showed the lowest membrane peroxidation level at harvest and during storage. No decay was recorded for 28 days of storage with the application of SA or chitosan. The upregulation of ripening-related *PaACS1* gene was also more correlated to the higher concentrations of AVG, followed by SA and then chitosan. Overall, AVG treatment led to an uncommon green color, along with high rates of WL and DI during cold storage, which make the fruit unmarketable. Therefore, this study suggests the application of SA (4 mM) at 30 and 15 days before harvest for optimal yield, quality, and storability of ‘Canino’ apricot fruit grown under Egyptian conditions. Future research could include the impact of ethylene inhibitors at all three stages of fruit growth and development, as well as studying more ripening-related genes associated with ethylene biosynthesis.

## Figures and Tables

**Table 1:** Effect of preharvest foliar application of AVG, SA, and chitosan on fruit color (L* “lightness”, a*” red/green”, and b* “yellow/blue”) of ‘Canino’ apricot at harvest date, and after 28 days of storage at 0 °C and 90% RH, followed by 2 days of shelf life at 20 ± 2 °C and 80–85% RH during the 2019 and 2020 seasons. Total color difference (ΔE) represents the change in color after 28 days.

Treatment	Harvest Date			28-Day Storage			ΔE
	L*	a*	b*	L*	a*	b*	
**Season 2019**							
Control	56.90a ± 0.83	4.37a ± 0.07	42.22a ± 0.10	65.20e ± 0.22	14.32a ± 0.07	48.43a ± 0.46	14.39g ± 0.51
AVG-a	41.85e ± 0.46	−10.0g ± 0.20	30.00f ± 0.20	68.52d ± 0.46	2.00g ± 0.08	35.59e ± 0.76	29.78d ± 0.27
AVG-b	50.38d ± 0.32	−8.36f ± 0.15	32.48e ± 0.06	68.77d ± 0.33	8.23f ± 0.07	37.66d ± 1.16	25.32e ± 0.29
SA-a	52.86c ± 0.67	−6.57e ± 0.07	34.77d ± 0.36	85.63a ± 0.84	10.45e ± 0.16	44.44d ± 0.53	38.18a ± 0.27
SA-b	53.7bc ± 0.40	−4.95d ± 0.06	36.08t ± 0.09	82.08b ± 0.87	10.91d ± 0.15	47.33a ± 0.34	34.40b ± 0.58
Chitosan-a	54.64b ± 0.13	1.44c ± 0.11	35.02d ± 0.18	85.74a ± 0.36	12.72c ± 0.09	39.87c ± 1.34	33.45c ± 0.39
Chitosan-b	56.42a ± 0.44	2.22b ± 0.11	37.70b ± 0.20	72.43c ± 0.12	13.49b ± 0.19	45.45b ± 1.36	21.08f ± 0.14
**Season 2020**							
Control	62.59a ± 0.91	4.73a ± 0.10	38.36a ± 0.10	74.51e ± 0.15	13.20a ± 0.01	47.95a ± 0.45	17.51e ± 0.47
AVG-a	46.04e ± 0.50	−10.0g ± 0.14	28.59e ± 0.46	75.03e ± 0.18	1.90g ± 0.07	35.09f ± 0.04	32.07b ± 0.77
AVG-b	55.41d ± 0.35	−8.74f ± 0.11	30.32d ± 0.69	77.25d ± 0.38	7.68f ± 0.24	37.81e ± 0.70	28.33c ± 0.71
SA-a	58.15c ± 0.73	−7.25e ± 0.07	35.78b ± 0.12	89.92a ± 0.88	9.70e ± 0.03	44.00c ± 0.53	36.93a ± 0.30
SA-b	59.08bc ± 0.44	−5.49d ± 0.06	37.93a ± 0.16	86.18b ± 0.91	9.94d ± 0.08	46.86ab ± 0.34	32.44b ± 0.69
Chitosan-a	60.10b ± 0.15	1.63c ± 0.06	32.21c ± 0.67	90.02a ± 0.38	11.74c ± 0.05	39.47d ± 1.33	32.41b ± 0.41
Chitosan-b	62.06a ± 0.48	2.53b ± 0.04	36.48b ± 0.03	78.91c ± 0.12	12.56b ± 0.04	46.18b ± 0.06	21.88d ± 0.35

Control = distilled water, AVG-a = 150 mg·L^−1^, AVG-b = 100 mg·L^−1^, SA-a = 4 mM, SA-b = 2 mM, Chitosan-a = 2.5%, and Chitosan-b = 1.5%. Values are the mean ± SD. Means followed by the same letter within a column are not significantly different using DMRT at *p* ≤ 0.05.

**Table 2:** Effect of preharvest foliar application of AVG, SA, and chitosan on the soluble solid content (SSC) of ‘Canino’ apricot fruit at harvest (0 day), and after 7, 14, 21, and 28 days of storage at 0 °C and 90% RH, followed by 2 days of shelf life at 20 ± 2 °C and 80–85% RH during the 2019 and 2020 seasons.

Treatment	SSC (%)				
	0 Day	7 Days	14 Days	21 Days	28 Days
**Season 2019**					
Control	11.46a ± 0.01	12.50a ± 0.15	12.93a ± 0.12	13.84a ± 0.15	14.82a ± 0.08
AVG-a	7.80f ± 0.10	9.77f ± 0.09	10.10e ± 0.05	10.26g ± 0.04	11.36f ± 0.24
AVG-b	8.12e ± 0.08	9.97 e ± 0.12	11.16d ± 0.01	11.71f ± 0.14	12.13e ± 0.07
SA-a	9.28d ± 0.18	11.03c ± 0.06	11.32d ± 0.12	12.40d ± 0.10	13.32d ± 0.08
SA-b	10.72b ± 0.13	11.17c ± 0.01	11.38d ± 0.18	12.87b ± 0.12	14.13b ± 0.07
Chitosan-a	9.82c ± 0.07	10.81d ± 0.04	11.70c ± 0.15	11.90e ± 0.02	13.82c ± 0.03
Chitosan-b	10.63b ± 0.09	11.59b ± 0.06	12.07b ± 0.12	12.64c ± 0.11	14.20b ± 0.10
**Season 2020**					
Control	10.96a ± 0.11	11.85a ± 0.11	12.32a ± 0.08	12.93a ± 0.10	14.82a ± 0.08
AVG-a	7.28f ± 0.02	9.87 e ± 0.08	10.02f ± 0.07	11.07d ± 0.18	11.26e ± 0.05
AVG-b	7.78e ± 0.12	10.89c ± 0.09	11.17e ± 0.01	11.71c ± 0.09	12.09d ± 0.11
SA-a	8.80d ± 0.10	10.59d ± 0.18	11.39d ± 0.14	11.74c ± 0.07	12.50d ± 0.13
SA-b	9.25c ± 0.12	11.40b ± 0.07	12.14b ± 0.02	12.90a ± 0.10	14.14b ± 0.10
Chitosan-a	10.48b ± 0.11	11.33b ± 0.17	11.40d ± 0.05	12.42b ± 0.12	13.66c ± 0.66
Chitosan-b	10.78a ± 0.18	11.47b ± 0.01	11.76c ± 0.09	12.57b ± 0.28	14.22b ± 0.10

Control = distilled water, AVG-a = 150 mg·L^−1^, AVG-b = 100 mg·L^−1^, SA-a = 4 mM, SA-b = 2 mM, Chitosan-a = 2.5%, and Chitosan-b = 1.5%. Values are the mean ± SD. Means followed by the same letter within a column are not significantly different using DMRT at *p* ≤ 0.05.

**Table 3:** Effect of preharvest foliar application of AVG, SA, and chitosan on the total acidity (TA) content of ‘Canino’ apricot fruit at harvest (0 day), and after 7, 14, 21, and 28 days of storage at 0 °C and 90% RH, followed by 2 days of shelf life at 20 ± 2 °C and 80–85% RH during the 2019 and 2020 seasons.

Treatment	TA (%)				
	0 Day	7 Days	14 Days	21 Days	28 Days
**Season 2019**					
Control	2.10e ± 0.03	1.31e ± 0.02	1.30g ± 0.02	1.08e ± 0.01	0.68f ± 0.01
AVG-a	2.59a ± 0.05	2.32a ± 0.03	2.27a ± 0.05	2.15a ± 0.03	1.50a ± 0.02
AVG-b	2.43bc ± 0.04	2.12b ± 0.02	1.96c ± 0.01	1.78b ± 0.05	1.17c ± 0.02
SA-a	2.47b ± 0.05	2.13b ± 0.01	2.06b ± 0.03	1.87b ± 0.04	1.34b ± 0.03
SA-b	2.31d ± 0.02	2.06c ± 0.01	1.70e ± 0.03	1.41c ± 0.01	1.07d ± 0.01
Chitosan-a	2.36cd ± 0.04	2.09bc ± 0.03	1.78d ± 0.01	1.42c ± 0.10	1.17c ± 0.03
Chitosan-b	2.12e ± 0.01	1.95d ± 0.03	1.37f ± 0.00	1.31d ± 0.03	0.85e ± 0.02
**Season 2020**					
Control	1.97c ± 0.02	1.29d ± 0.00d	1.22d ± 0.01	0.99e ± 0.02	0.66e ± 0.03
AVG-a	2.37a ± 0.05	2.30a ± 0.01a	2.25a ± 0.00	1.77a ± 0.03	1.41a ± 0.06
AVG-b	2.27ab ± 0.00	1.94b ± 0.06b	1.87b ± 0.01	1.70b ± 0.01	1.37a ± 0.04
SA-a	2.34ab ± 0.01	1.94b ± 0.12b	1.88b ± 0.07	1.78a ± 0.01	1.01b ± 0.02
SA-b	2.08c ± 0.01	1.83c ± 0.05c	1.70bc ± 0.02	1.32cd ± 0.10	0.91c ± 0.01
Chitosan-a	2.22b ± 0.19	1.93bc ± 0.03	1.70bc ± 0.01	1.37c ± 0.02	0.97b ± 0.04
Chitosan-b	2.07c ± 0.01	1.88bc ± 0.01	1.56c ± 0.37	1.27d ± 0.03	0.82d ± 0.05

Control = distilled water, AVG-a = 150 mg·L^−1^, AVG-b = 100 mg·L^−1^, SA-a = 4 mM, SA-b = 2 mM, Chitosan-a = 2.5%, and Chitosan-b = 1.5%. Values are the mean ± SD. Means followed by the same letter within a column are not significantly different using DMRT at *p* ≤ 0.05.

**Table 4:** Effect of preharvest foliar application of AVG, SA, and chitosan on the ripening index (RI) of ‘Canino’ apricot fruit at harvest (0 day), and after 7, 14, 21, and 28 days of storage at 0 °C and 90% RH, followed by 2 days of shelf life at 20 ± 2 °C and 80–85% RH during the 2019 and 2020 seasons.

Treatment	RI (SSC/TA)				
	0 Day	7 Days	14 Days	21 Days	28 Days
**Season 2019**					
Control	5.45a ± 0.08	9.57a ± 0.14	9.97a ± 0.25	12.77a ± 0.04	21.90a ± 0.34
AVG-a	3.01g ± 0.04	4.22f ± 0.08	4.45e ± 0.12	4.77e ± 0.08	7.60g ± 0.12
AVG-b	3.34f ± 0.11	4.69e ± 0.04	5.68d ± 0.02	6.57d ± 0.25	10.38e ± 0.20
SA-a	3.76e ± 0.03	5.19d ± 0.05	5.49d ± 0.13	6.65d ± 0.21	9.94f ± 0.22
SA-b	4.64c ± 0.09	5.41c ± 0.01	6.69c ± 0.20	9.15b ± 0.11	13.25c ± 0.17
Chitosan-a	4.17d ± 0.07	5.16d ± 0.07	6.56c ± 0.11	8.43c ± 0.59	11.86d ± 0.30
Chitosan-b	5.02b ± 0.07	5.94b ± 0.08	8.81b ± 0.11	9.64b ± 0.17	16.69b ± 0.28
**Season 2020**					
Control	5.58a ± 0.05	9.21a ± 0.09	9.58a ± 0.06	13.03a ± 0.18	22.38a ± 1.19
AVG-a	3.07g ± 0.05	4.29e ± 0.05	4.35e ± 0.02	6.24e ± 0.03	7.97f ± 0.32
AVG-b	3.43f ± 0.06	5.62d ± 0.14	5.76d ± 0.18	6.91d ± 0.07	8.85f ± 0.23
SA-a	3.77e ± 0.05	5.46d ± 0.29	5.88d ± 0.30	6.58de ± 0.09	12.41e ± 0.27
SA-b	4.45d ± 0.05	6.23b ± 0.13	6.64b ± 0.18	9.78b ± 0.67	15.61c ± 0.18
Chitosan-a	4.74c ± 0.39	5.89c ± 0.13	5.92d ± 0.08	9.04c ± 0.11	14.05d ± 1.17
Chitosan-b	5.20b ± 0.06	6.11bc ± 0.02	6.27c ± 0.07	9.89b ± 0.43	17.35b ± 1.09

Control = distilled water, AVG-a = 150 mg·L^−1^, AVG-b = 100 mg·L^−1^, SA-a = 4 mM, SA-b = 2 mM, Chitosan-a = 2.5%, and Chitosan-b = 1.5%. Values are the mean ± SD. Means followed by the same letter within a column are not significantly different using DMRT at *p* ≤ 0.05.

**Table 5:** Soil analysis of the experimental site.

Chemical Characteristic	Value
EC (ds·m^−1^)	1.45
pH	7.93
CaCO_3_ (%)	8.54
CO_3_^−^	0.00
HCO_3_^−^	0.90
Cl^−^	0.50
SO_4_^−2^	0.26
K^+^	0.21
Mg^+2^	0.20
Na^+^	0.45
Ca^+2^	0.80

## Data Availability

Not applicable.

## References

[1] Hallmann E., Rozpara E., Słowianek M., Leszczyńska J. (2019). The effect of organic and conventional farm management on the allergenic potency and bioactive compounds status of apricots *(Prunus armeniaca* L.). Food Chem..

[2] Liu J., Deng J.L., Tian Y. (2020). Transcriptome sequencing of the apricot *(Prunus armeniaca* L.) and identification of differentially expressed genes involved in drought stress. Phytochemistry.

[3] Food and Agriculture Organization of the United Nations (FAO) (2019). FAO Statistics.

[4] Taze B.H., Unluturk S. (2018). Effect of postharvest UV-C treatment on the microbial quality of ‘Şalak’ apricot. Sci. Hortic..

[5] Okba S.K., Mazrou Y., Elmenofy H.M., Ezzat A., Salama A. (2021). New insights of Potassium Sources Impacts as Foliar Applica-tion on “Canino” Apricot Fruit Yield, Fruit Anatomy, Quality and Storability. Plants.

[6] Nourozi F., Sayyari M. (2020). Enrichment of Aloe vera gel with basil seed mucilage preserve bioactive compounds and postharvest quality of apricot fruits. Sci. Hortic..

[7] Bravin E., Kilchenmann A., Leumann M. (2009). Six hypotheses for profitable apple production based on the economic work-package within the ISAFRUIT Project. J. Hortic. Sci. Biotechnol..

[8] Fan X., Shu C., Zhao K., Wang X., Cao J., Jiang W. (2018). Regulation of apricot ripening and softening process during shelf life by post-storage treatments of exogenous ethylene and 1-methylcyclopropene. Sci. Hortic..

[9] He Y., Xue J., Li H., Han S., Jiao J., Rao J. (2020). Ethylene response factors regulate ethylene biosynthesis and cell wall modification in persimmon (*Diospyros kaki* L.) fruit during ripening. Postharvest Biol. Technol..

[10] Kou J., Zhao Z., Zhang Q., Wei C., Ference C.M., Guan J., Wang W. (2021). Comparative transcriptome analysis reveals the mechanism involving ethylene and cell wall modification related genes in Diospyros kaki fruit firmness during ripening. Genomics.

[11] Devlieghere F., Jacxsens L., Serna Tatay M., Debevere J., Meirlaen J., Vanrolleghem P. (2003). Modelling the relation between ethylene production rate, respiration rate and their influence on climacteric and non-climacteric fruits. Acta Hortic..

[12] Ozkan Y., Ozturk B., Yildiz K. (2016). Effects of aminoethoxyvinylglycine and naphthaleneacetic acid on ethylene biosynthesis, pre-harvest fruit drop and fruit quality of apple. Pakistan J. Agric. Sci..

[13] Yildiz K., Kilic K., Ozkan Y., Ozturk B., Kucuker E. (2018). The role of Pre-harvest Aminoethoxyvinylglycine (AVG) Treatments on Total Phenolics, Antioxidant Capacity and Fruit Quality Attributes of Sweet Cherry Cultivars. Erwerbs-Obstbau.

[14] Lin Z., Zhong S., Grierson D. (2009). Recent advances in ethylene research. J. Exp. Bot..

[15] Yamagami T., Tsuchisaka A., Yamada K., Haddon W.F., Harden L.A., Theologis A. (2003). Biochemical diversity among the 1-aminocyclopropane-1-carboxylate synthase isozymes encoded by the Arabidopsis gene family. J. Biol. Chem..

[16] El-Sharkawy I., Kim W.S., Jayasankar S., Svircev A.M., Brown D.C.W. (2008). Differential regulation of four members of the ACC synthase gene family in plum. J. Exp. Bot..

[17] Munoz-Roberdo P., Rubio P., Infante R., Campos-Vargas R., Manriquez D., Gonzalez-Aguero M., Defillipi B.G. (2012). Ethylene biosynthesis in apricot: Identification of ripening-related 1-aminocyclopropane-1-carboxylic acid synthase (*ACS*) gene. Postharvest Biol. Technol..

[18] Tarantino A., Lops F., Disciglio G., Lopriore G. (2018). Effects of plant biostimulants on fruit set, growth, yield and fruit quality attributes of ‘Orange rubis^®^’ apricot (*Prunus armeniaca* L.) cultivar in two consecutive years. Sci. Hortic..

[19] Huo K., Shui L., Mai Y., Zhou N., Liu Y., Zhang C., Niu J. (2020). Effects of exogenous abscisic acid on oil content, fatty acid composition, biodiesel properties and lipid components in developing Siberian apricot (*Prunus sibirica*) seeds. Plant Physiol. Biochem..

[20] Cui K., Shu C., Zhao H., Fan X., Cao J., Jiang W. (2020). Preharvest chitosan oligochitosan and salicylic acid treatments enhance phenol metabolism and maintain the postharvest quality of apricots (*Prunus armeniaca* L.). Sci. Hortic..

[21] Batur S., Çetinbaş M. (2017). Pre-harvest Application of ReTain (Aminoethoxyvinylglycine, AVG) Influences Pre-harvest Drop and Fruit Quality of ‘Williams’ Pears. Tarım Bilim. Derg..

[22] Doerflinger F.C., Nock J.F., Miller W.B., Watkins C.B. (2019). Preharvest aminoethoxyvinylglycine (AVG) and 1-methylcyclopropene (1-MCP) effects on ethylene and starch concentrations of ‘Empire’ and ‘McIntosh’ apples. Sci. Hortic..

[23] Gerailoo S., Ghasemnezhad M. (2011). Effect of Salicylic Acid on Antioxidant Enzyme and Petal Senescence in ‘Yellow Island’ Cut Rose Flowers. J. Fruit Ornam. Plant Res..

[24] Zhang H., Ma Z., Wang J., Wang P., Lu D., Deng S., Lei H., Gao Y., Tao Y. (2021). Treatment with exogenous salicylic acid maintains quality, increases bioactive compounds, and enhances the antioxidant capacity of fresh goji (*Lycium barbarum* L.) fruit during storage. LWT.

[25] Tezotto-Uliana J.V., Fargoni G.P., Geerdink G.M., Kluge R.A. (2014). Chitosan applications pre- or postharvest prolong raspberry shelf-life quality. Postharvest Biol. Technol..

[26] Peian Z., Haifeng J., Peijie G., Sadeghnezhad E., Qianqian P., Tianyu D., Teng L., Huanchun J., Jinggui F. (2021). Chitosan induces jasmonic acid production leading to resistance of ripened fruit against *Botrytis cinerea* infection. Food Chem..

[27] Iqbal S., Ni X., Bilal M.S., Shi T., Khalil-ur-Rehman M., Zhenpeng P., Jie G., Usman M., Gao Z. (2020). Identification and expression profiling of sugar transporter genes during sugar accumulation at different stages of fruit development in apricot. Gene.

[28] García-Gómez B.E., Ruiz D., Salazar J.A., Rubio M., Martínez-García P.J., Martínez-Gómez P. (2020). Analysis of Metabolites and Gene Expression Changes Relative to Apricot (*Prunus armeniaca* L.) Fruit Quality during Development and Ripening. Front. Plant Sci..

[29] Salazar J., Zapata P., Silva C., González M., Pacheco I., Bastías M., Meneses C., Jorquera C., Moreno I., Shinya P. (2021). Transcriptome analysis and postharvest behavior of the kiwifruit ‘*Actinidia deliciosa*’ reveal the role of ethylene-related phytohormones during fruit ripening. Tree Genet. Genomes.

[30] Yang R., Lin X., Dou Y., Zhang W., Du H., Wan C., Chen J., Zhang L., Zhu L. (2021). Transcriptome profiling of postharvest kiwifruit in response to exogenous nitric oxide. Sci. Hortic..

[31] Chen J., Chen T., Qiu M., Li L., Zhong Q., Wei Q., Deng Y., Xie B., Jiang Y., Chen B. (2021). Identification of ACC synthetase genes in *Volvariella volvacea* and analysis of their response to ethephon and 1-methylcyclopropene treatments. Sci. Hortic..

[32] Yumbya P., Ambuko J., Hutchinson M., Owino W., Juma J., Machuka E., Mutuku J.M. (2021). Transcriptome analysis to elucidate hexanal’s mode of action in preserving the post-harvest shelf life and quality of banana fruits (*Musa acuminata*). J. Agric. Food Res..

[33] García-Gómez B.E., Salazar J.A., Nicolás-Almansa M., Razi M., Rubio M., Ruiz D., Martínez-Gómez P. (2021). Molecular Bases of Fruit Quality in Prunus Species: An Integrated Genomic, Transcriptomic, and Metabolic Review with a Breeding Perspective. Int. J. Mol. Sci..

[34] D’Aquino S., Schirra M., Molinu M.G., Tedde M., Palma A. (2010). Preharvest aminoethoxyvinylglycine treatments reduce internal browning and prolong the shelf-life of early ripening pears. Sci. Hortic..

[35] Radwa F.S., Attia M.M., Hassan A.K., Yehia S.M. (2019). Effect of Postharvest Aminoethoxyvinylglycine, 1-Methylcyclopropene and Jasmonic Acid Treatments on Storability and Quality Maintenance of Apricot Fruit Cv. “Canino.” Alexandria J. Agric. Sci..

[36] Hatem R.M.K. (2019). Effect of some Preharvest Treatments on Fruit Drop, Quality and Shelf Life of “Anna” Apple Fruits. J. Plant Prod..

[37] Muzzaffar S., Bhat M.M., Wani T.A., Wani I.A., Masoodi F.A., Mir S., Shah M., Mir M. (2018). Postharvest biology and technology of apricot. Postharvest Biology and Technology of Temperate Fruits.

[38] Pokotylo I., Kravets V., Ruelland E. (2019). Salicylic acid binding proteins (SABPs): The hidden forefront of salicylic acid signalling. Int. J. Mol. Sci..

[39] Pérez-Llorca M., Muñoz P., Müller M., Munné-Bosch S. (2019). Biosynthesis, metabolism and function of auxin, salicylic acid and melatonin in climacteric and non-climacteric fruits. Front. Plant Sci..

[40] Lokesh G., Madhumathi C., Rama Krishna M., Tanuja Priya B., Kadiri L. (2020). Influence of preharvest application of salicylic acid and potassium silicate on postharvest quality of mango fruits (*Mangifera indica* L.) cv. Alphonso. Acta Sci. Agric..

[41] Loake G., Grant M. (2007). Salicylic acid in plant defence—The players and protagonists. Curr. Opin. Plant Biol..

[42] Arif Y., Sami F., Siddiqui H., Bajguz A., Hayat S. (2020). Salicylic acid in relation to other phytohormones in plant: A study towards physiology and signal transduction under challenging environment. Environ. Exp. Bot..

[43] Leslie C.A., Romani R.J. (1988). Inhibition of ethylene biosynthesis by salicylic acid. Plant Physiol..

[44] Da Rocha Neto A.C., Luiz C., Maraschin M., Di Piero R.M. (2016). Efficacy of salicylic acid to reduce Penicillium expansum inoculum and preserve apple fruits. Int. J. Food Microbiol..

[45] Serrano M., Giménez M.J., Martínez-Esplá A., Valverde J.M., Martinez-Romero D., Castillo S., Valero D. (2018). Effects of preharvest salicylate treatments on quality and antioxidant compounds of plums. Acta Hortic..

[46] Martínez-Esplá A., Zapata P.J., Valero D., Martínez-Romero D., Díaz-Mula H.M., Serrano M. (2018). Preharvest treatments with salicylates enhance nutrient and antioxidant compounds in plum at harvest and after storage. J. Sci. Food Agric..

[47] Giménez M.J., Serrano M., Valverde J.M., Martínez-Romero D., Castillo S., Valero D., Guillén F. (2017). Preharvest salicylic acid and acetylsalicylic acid treatments preserve quality and enhance antioxidant systems during postharvest storage of sweet cherry cultivars. J. Sci. Food Agric..

[48] Mansour A.H.A., Elmenofy H.M., Salama A.-M. (2020). Effect of Preharvest Application of Some Antioxidants on The Fruit Yield, Quality and Storability of “Manfalouty” Pomegranate Fruits (*Punica granatum* L.). Middle East J. Agric. Res..

[49] Ezzat A., Ammar A., Szabó Z., Nyéki J., Holb I.J. (2017). Postharvest Treatments with Methyl Jasmonate and Salicylic Acid for Maintaining Physico-Chemical Characteristics and Sensory Quality Properties of Apricot Fruit during Cold Storage and Shelf-Life. Pol. J. Food Nutr. Sci..

[50] Ezzat A., Hegedűs A., Szabó S., Ammar A., Szabó Z., Nyéki J., Molnár B., Holb I.J. (2020). Temporal changes and correlations between quality loss parameters, antioxidant properties and enzyme activities in apricot fruit treated with methyl jasmonate and salicylic acid during cold storage and shelf-life. Appl. Sci..

[51] Batool M., Bashir O., Amin T., Wani S.M., Masoodi F.A., Jan N., Bhat S.A., Gul A. (2021). Investigating the effect of oxalic acid and salicylic acid treatments on the post-harvest life of temperate grown apricot varieties (*Prunus armeniaca*) during controlled atmosphere storage. Food Sci. Technol. Int..

[52] Sharif R., Mujtaba M., Rahman M.U., Shalmani A., Ahmad H., Anwar T., Tianchan D., Wang X. (2018). The multifunctional role of in horticultural crops; a review. Molecules.

[53] Gull A., Bhat N., Wani S.M., Masoodi F.A., Amin T., Ganai S.A. (2021). Shelf life extension of apricot fruit by application of nanochitosan emulsion coatings containing pomegranate peel extract. Food Chem..

[54] Baswal A.K., Dhaliwal H.S., Singh Z., Mahajan B.V.C., Kalia A., Gill K.S. (2020). Influence of carboxy methylcellulose, chitosan and beeswax coatings on cold storage life and quality of Kinnow mandarin fruit. Sci. Hortic..

[55] Cindi M.D., Shittu T., Sivakumar D., Bautista-Baños S. (2015). Chitosan boehmite-alumina nanocomposite films and thyme oil vapour control brown rot in peaches (*Prunus persica* L.) during postharvest storage. Crop Prot..

[56] Zhao H., Fan Z., Wu J., Zhu S. (2021). Effects of pre-treatment with S-nitrosoglutathione-chitosan nanoparticles on quality and antioxidant systems of fresh-cut apple slices. LWT.

[57] Munhuweyi K., Lennox C.L., Meitz-Hopkins J.C., Caleb O.J., Sigge G.O., Opara U.L. (2017). Investigating the effects of crab shell chitosan on fungal mycelial growth and postharvest quality attributes of pomegranate whole fruit and arils. Sci. Hortic..

[58] Elmenofy H.M., Mark C. (2021). Effect of Natural Antimicrobial Substances with Packaging System on Improving Quality of ‘ETMANI’ Guava (*Psidium guajava* L.) Fruit during cold storage. J. Plant Prod..

[59] Arseneault M.H., Cline J.A. (2016). A review of apple preharvest fruit drop and practices for horticultural management. Sci. Hortic..

[60] Souza K.O., Silveira A.G., Lopes M.M.A., Moura C.F.H., Silva E.O., Fernando Ayala-Zavala J., Soares L.S.P., Miranda M.R.A. (2019). AVG and GA_3_ prevent preharvest fruit drop and enhance postharvest quality of “BRS 189” cashew. Sci. Hortic..

[61] Arseneault M.H., Cline J.A. (2017). AVG, NAA, boron, and magnesium influence preharvest fruit drop and fruit quality of ‘Honeycrisp’ apples. Can. J. Plant Sci..

[62] Gomes E.P., Vanz Borges C., Monteiro G.C., Filiol Belin M.A., Minatel I.O., Pimentel Junior A., Tecchio M.A., Lima G.P.P. (2021). Preharvest salicylic acid treatments improve phenolic compounds and biogenic amines in ‘Niagara Rosada’ table grape. Postharvest Biol. Technol..

[63] Wu P., Xin F., Xu H., Chu Y., Du Y., Tian H., Zhu B. (2021). Chitosan inhibits postharvest berry abscission of ‘Kyoho’ table grapes by affecting the structure of abscission zone, cell wall degrading enzymes and SO_2_ permeation. Postharvest Biol. Technol..

[64] Hou Y., Wu F., Zhao Y., Shi L., Zhu X. (2019). Cloning and expression analysis of polygalacturonase and pectin methylesterase genes during softening in apricot (*Prunus armeniaca* L.) fruit. Sci. Hortic..

[65] Parven A., Sarker M.R., Megharaj I.M. (2020). Meftaul, I. Prolonging the shelf life of Papaya (*Carica papaya* L.) using Aloe vera gel at ambient temperature. Sci. Hortic..

[66] Jongsri P., Wangsomboondee T., Rojsitthisak P., Seraypheap K. (2016). Effect of molecular weights of chitosan coating on postharvest quality and physicochemical characteristics of mango fruit. LWT-Food Sci. Technol..

[67] Coggins C.W., Scora R.W., Lewis L.N., Knapp C.F. (1969). Gibberellin-delayed senescence and essential oil vhanges in the Navel orange rind. J. Agric. Food Vhem..

[68] El-Otmani M., Wardowski W.E., Miller W.M., Hall D.J., Grierson W. (2006). Growth regulator improvement of postharvest quality. Fresh Citrus Fruits.

[69] Eckert J.W., Eaks I.L., Reuther W., Calavan E.C., Carman G.E. (1989). Postharvest disorders and diseases of citrus fruits. The Citus Industry.

[70] Smilanick J.L., Brown G.E., Eckert J.W., Wardowski W.E., Miller W.M., Hall D.J., Grierson W. (2006). The biology and control of postharvest diseases. Fresh Citrus Fruits.

[71] De Vleesschauwer D., Seifi H.S., Filipe O., Haeck A., Huu S.N., Demeestere K., Höfte M. (2016). The DELLA protein SLR1 integrates and amplifies salicylic acid- and jasmonic acid-dependent innate immunity in rice. Plant Physiol..

[72] Ahmad S., Singh Z., Khan A.S., Iqbal Z. (2013). Preharvest applications of salicylic acid maintain the rind textural properities and reduce fruit rot and chilling injury of sweet orange during cold storage. Pak. J. Agric. Sci..

[73] Shemy M.A. (2020). El Effect of some essential oils, salts and salicylic acid on reducing decay, keeping quality and prolonging shelf-life of canino apricot fruits. Menoufia J. Plant Prod..

[74] De Oliveira C.E.V., Magnani M., de Sales C.V., de Souza Pontes A.L., Campos-Takaki G.M., Stamford T.C.M., de Souza E.L. (2014). Effects of chitosan from Cunninghamella elegans on virulence of post-harvest pathogenic fungi in table grapes (*Vitis labrusca* L.). Int. J. Food Microbiol..

[75] Ma Z., Yang L., Yan H., Kennedy J.F., Meng X. (2013). Chitosan and oligochitosan enhance the resistance of peach fruit to brown rot. Carbohydr. Polym..

[76] Romanazzi G., Feliziani E., Baños S.B., Sivakumar D. (2017). Shelf life extension of fresh fruit and vegetables by chitosan treatment. Crit. Rev. Food Sci. Nutr..

[77] Xing Y., Xu Q., Yang S.X., Chen C., Tang Y., Sun S., Zhang L., Che Z., Li X. (2016). Preservation mechanism of chitosan-based coating with cinnamon oil for fruits storage based on sensor data. Sensors.

[78] Xu D., Qin H., Ren D. (2018). Prolonged preservation of tangerine fruits using chitosan/montmorillonite composite coating. Postharvest Biol. Technol..

[79] Cai C., Ma R., Duan M., Deng Y., Liu T., Lu D. (2020). Effect of starch film containing thyme essential oil microcapsules on physicochemical activity of mango. LWT.

[80] Arroyo B.J., Bezerra A.C., Oliveira L.L., Arroyo S.J., de Melo E.A., Santos A.M.P. (2020). Antimicrobial active edible coating of alginate and chitosan add ZnO nanoparticles applied in guavas (*Psidium guajava* L.). Food Chem..

[81] Ayala-Silva T., Schnell R.J., Meerow A.W., Winterstein M., Cervantes C., Brown J.S. (2005). Determination of color and fruit traits of half-sib families of mango (*Mangifers indica* L.). Proc. Fla. State Hort. Soc..

[82] Zhou W., Niu Y., Ding X., Zhao S., Li Y., Fan G., Zhang S., Liao K. (2020). Analysis of carotenoid content and diversity in apricots (*Prunus armeniaca* L.) grown in China. Food Chem..

[83] Valdés H., Pizarro M., Campos-Vargas R., Infante R., Defilippi B.G. (2009). Effect of ethylene inhibitors on quality attributes of apricot cv. Modesto and Patterson during Storage. Chil. J. Agric. Res..

[84] Ruiz D., Egea J., Tomás-Barberán F.A., Gil M.I. (2005). Carotenoids from new apricot (*Prunus armeniaca* L.) varieties and their relationship with flesh and skin color. J. Agric. Food Chem..

[85] Intrigliolo D.S., Castel J.R. (2010). Response of plum trees to deficit irrigation under two crop levels: Tree growth, yield and fruit quality. Irrig. Sci..

[86] Batista-Silva W., Nascimento V.L., Medeiros D.B., Nunes-Nesi A., Ribeiro D.M., Zsögön A., Araújo W.L. (2018). Modifications in organic acid profiles during fruit development and ripening: Correlation or causation?. Front. Plant Sci..

[87] Stanley J., Prakash R., Marshall R., Schröder R. (2013). Effect of harvest maturity and cold storage on correlations between fruit properties during ripening of apricot (*Prunus armeniaca*). Postharvest Biol. Technol..

[88] Dragovic-Uzelac V., Levaj B., Mrkic V., Bursac D., Boras M. (2007). The content of polyphenols and carotenoids in three apricot cultivars depending on stage of maturity and geographical region. Food Chem..

[89] Balasundram N., Sundram K., Samman S. (2006). Phenolic compounds in plants and agri-industrial by-products: Antioxidant activity, occurrence, and potential uses. Food Chem..

[90] Gao H., Zhang Z.K., Chai H.K., Cheng N., Yang Y., Wang D.N., Yang T., Cao W. (2016). Melatonin treatment delays postharvest senescence and regulates reactive oxygen species metabolism in peach fruit. Postharvest Biol. Technol..

[91] Wang Q.J., Sun H., Dong Q.L., Sun T.Y., Jin Z.X., Hao Y.J., Yao Y.X. (2016). The enhancement of tolerance to salt and cold stresses by modifying the redox state and salicylic acid content via the cytosolic malate dehydrogenase gene in transgenic apple plants. Plant Biotechnol. J..

[92] Adiletta G., Pasquariello M.S., Zampella L., Mastrobuoni F., Scortichini M., Petriccione M. (2018). Chitosan coating: A Postharvest treatment to delay oxidative stress in loquat fruits during cold storage. Agronomy.

[93] Cetinbas M., Butar S., Onursal C.E., Koyuncu M.A. (2012). The effects of pre-harvest ReTain [aminoethoxyvinylglycine (AVG)] application on quality change of ‘Monroe’ peach during normal and controlled atmosphere storage. Sci. Hortic..

[94] Karacali I. (2009). Preservation and marketing of horticultural products. Ege Univ. Fac. Agric. Publ..

[95] McGlasson W.B., Rath A.C., Legendre L. (2005). Preharvest application of aminoethoxyvinyleglycine (AVC) modifies harvest maturity abd cool storage life of ‘Arctic Snow’ nextarines. Postharvest Biol. Technol..

[96] Jemric T., Ivic D., Fruk G., Skutin Matijas H., Cvjetkovic B., Bupic M., Pavkovic B. (2011). Reduction of postharvest decay of peach and nectarine caused by monilinia laxa using hot water dipping. Food Biopocess Technol..

[97] Snedecor G.W., Cochran W.G. (1990). Statistical Methods.

[98] McGuire R.G. (1992). Reporting of objective color measurements. HortScience.

[99] Bhanushree L., Vasudeva K., Suresha G., Sadananda G., Mohamad Tayeebulla H., Halesh G. (2018). Influence of chitosan on postharvest behavior of papaya (*Carica papaya* L.) Fruits under different storage conditions. J. Pharmacogn. Phytochem..

[100] Wellburn A.R. (1994). The Spectral Determination of Chlorophylls a and b, as well as Total Carotenoids, Using Various Solvents with Spectrophotometers of Different Resolution. J. Plant Physiol..

[101] AOAC (2005). Official Method of Analysis.

[102] Jatoi M.A., Jurić S., Vidrih R., Vinceković M., Vuković M., Jemrić T. (2017). The effects of postharvest application of lecithin to improve storage potential and quality of fresh goji (*Lycium barbarum* L.) berries. Food Chem..

[103] Zhao H., Dai T., Jing Q., Jiang D., Cao W. (2007). Leaf senescence and grain filling affected by post-anthesis high temperatures in two different wheat cultivars. Plant Growth Regul..

[104] Fan X.J., Zhang B., Yan H., Feng J.T., Ma Z.Q., Zhang X. (2019). Effect of lotus leaf extract incorporated composite coating on the postharvest quality of fresh goji (*Lycium barbarum* L.) fruit. Postharvest Biol. Technol..

[105] El-Adawy M., El-Aziz M.A., El-Shazly K., Ali N.G., El-Magd M.A. (2018). Dietary propionic acid enhances antibacterial and immunomodulatory effects of oxytetracycline on Nile tilapia, *Oreochromis niloticus*. Environ. Sci. Pollut. Res..

[106] Rao X., Huang X., Zhou Z., Lin X. (2013). An improvement of the 2ˆ(–delta delta CT) method for quantitative real-time polymerase chain reaction data analysis. Biostat. Bioinforma. Biomath..

[107] Duncan D.B. (1955). Multiple ranges and multiple F test. Biometrics.

